# The role of mid-insula in the relationship between cardiac interoceptive attention and anxiety: evidence from an fMRI study

**DOI:** 10.1038/s41598-018-35635-6

**Published:** 2018-11-22

**Authors:** Yafei Tan, Dongtao Wei, Meng Zhang, Junyi Yang, Valentina Jelinčić, Jiang Qiu

**Affiliations:** 1 Faculty of Psychology, Southwest University, Chongqing, 400715 China; 2 Key Laboratory of Cognition and Personality (SWU), Ministry of Education, Chongqing, 400715 China; 3 Health Psychology, University of Leuven, Leuven, 3000 Belgium; 4 Department of Psychology, Xinxiang Medical University, Henan, 453003 China

## Abstract

Interoception refers to the perception of the internal bodily states. Recent accounts highlight the role of the insula in both interoception and the subjective experience of anxiety. The current study aimed to delve deeper into the neural correlates of cardiac interoception; more specifically, the relationship between interoception-related insular activity, interoceptive accuracy, and anxiety. This was done using functional magnetic resonance imaging (fMRI) in an experimental design in which 40 healthy volunteers focused on their heartbeat and anxious events. Interoceptive accuracy and anxiety levels were measured using the Heartbeat Perception Task and State Trait Anxiety Inventory, respectively. The results showed posterior, mid and anterior insular activity during cardiac interoception, whereas anxiety-related activation showed only anterior insular activity. Activation of the anterior insula when focused on cardiac interoception was positively correlated to state and trait anxiety levels, respectively. Moreover, the mid-insular activity during the cardiac attention condition not only related to individuals’ interoceptive accuracy but also to their levels of state and trait anxiety, respectively. These findings confirm that there are distinct neural representations of heartbeat attention and anxious experience across the insular regions, and suggest the mid-insula as a crucial link between cardiac interoception and anxiety.

## Introduction

Interoception refers to the phenomenological perception of the physiological state of the body, resulting from the multimodal integration of sensory input^1–3^. Research on interoception has seen an exponential growth in recent years because of its potential importance for emotional experience, self-regulation, decision-making, and self-awareness^4–9^. The cardiac interoception has received particular attention due to the simple and quick methods of measuring it^10^. Cardiac perception, however, is not a monolithic concept. There are multiple distinct facets of interoception, an idea first proposed by Ceunen, *et al*.^11^ and later also acknowledged and expanded on by other researchers^12^. What almost everyone agrees on is that measures of interoception that are not directly reflective of accuracy require a different label from measures that do reflect accuracy. For example, cardiac accuracy measures refer to procedures that compare actual and perceived heartbeats. In our current study, we use an additional measure that does not directly reflect accuracy and which we will refer to as ‘attention only’. We chose this label because participants were requested to attend to their heartbeat and rate its intensity afterwards, without actual detection of heartbeats while they were attending.

Neuroimaging in interoception research has surged recently, and serves to point toward a pivotal role of the insular cortex in the perception, integration, and representation of the sensory signals that shape the perception of the physiological state of our body^1,13–15^. Specifically, it was proposed that the sympathetic afferents from the body’s internal state arrive to the posterior granular and mid-dysgranular regions of the insular cortex through a lamina I spinothalamocortical pathway^1^. Furthermore, the anterior agranular insula may serve to integrate the information from the outside world and the internal body^2,16^. This hierarchical neural system of interoception fits the theories that the neural re-representation of the physiological state of the body provides a basis for the experience of emotions^17,18^. For instance, Zaki, *et al*.^19^ found an overlap of activity in the anterior insula during heartbeat detection tasks on the one hand, and self-report on the emotional response to video clips on the other. Recent accounts further highlight the role of the insula in processing autonomic activity as well as subjective experience of anxiety^20,21^. For example, Critchley, *et al*.^22^ reported activity of the anterior insula during the heartbeat detection task, correlating interoceptive accuracy and levels of anxiety.

In the literature on the neural correlates of cardiac interoception, one subset of functional magnetic resonance imaging (fMRI) studies preceding our own can be classified as belonging to our ‘attention only’ category. In several studies, cardiac perception was measured by having participants focus their attention on sensations from the heart without actual measures of how accurate this perception was^15,23–26^. These interoceptive attention only studies indicate that mid-insular activity is reflective of attending to the heartbeat. The conclusion from these studies is corroborated by several meta-analyses suggesting a particular role of the mid-insula in attending to interoceptive processes^10,14,27^. A different subset of studies using fMRI to find the neural correlates of cardiac perception directly measured interoceptive accuracy. In other words, these studies required participants to track or detect their heartbeats while measures of accuracy were obtained^7,19,22,28–31^. These studies found that the activity in the anterior insula was associated with cardiac interoception.

Of note is that heartbeat detection may be sensitive to bias as it involves actively keeping track of one’s perceived heartbeat, and that this cognitive load may be the reason it activates a more anterior area of the insula. Here, we argue that the heartbeat attention only task during functional neuroimaging may be a good way to minimize effects of cognitive load, in addition to being a better method to observe how insular activity reflects interoceptive but not cognitive relationships to anxiety^3^. Accuracy measures obtained outside of the neuroimaging sessions are still useful, but more so to profile participants according to their level of interoceptive accuracy.

The current study was conducted with the intention to further clarify neural correlates of cardiac interoception and to expand on the current functional neuroanatomical models of interoception and anxiety, along with furthering the knowledge needed for suitable interventions in anxiety disorders. To this end, participants underwent fMRI while performing a well-validated heartbeat attention only task in which participants simply focused on the sensation of their heartbeat^15,23–26^. The fMRI task also included an anxiety attention condition during which participants reflected on personally relevant anxious events. This task was included to test previous findings indicating that anxiety attention involves a more anterior part of the insula relative to interoceptive attention^15^. In addition, outside of the fMRI scanner, individual differences in the interoceptive accuracy and levels of state and trait anxiety were measured using the Heartbeat Perception Task^32^ and State Trait Anxiety Inventory^33^, respectively. Based on prior studies^15,23–26^, we hypothesized that the mid-insula would be active when attending to the heartbeat, while activity in the anterior insula would be most strongly expressed during anxiety attention. We further hypothesized that activity in the mid-insula during heartbeat attention would be correlated with both interoceptive accuracy and levels of state and trait anxiety.

## Results

### Behavioral data

**Figure 1:**
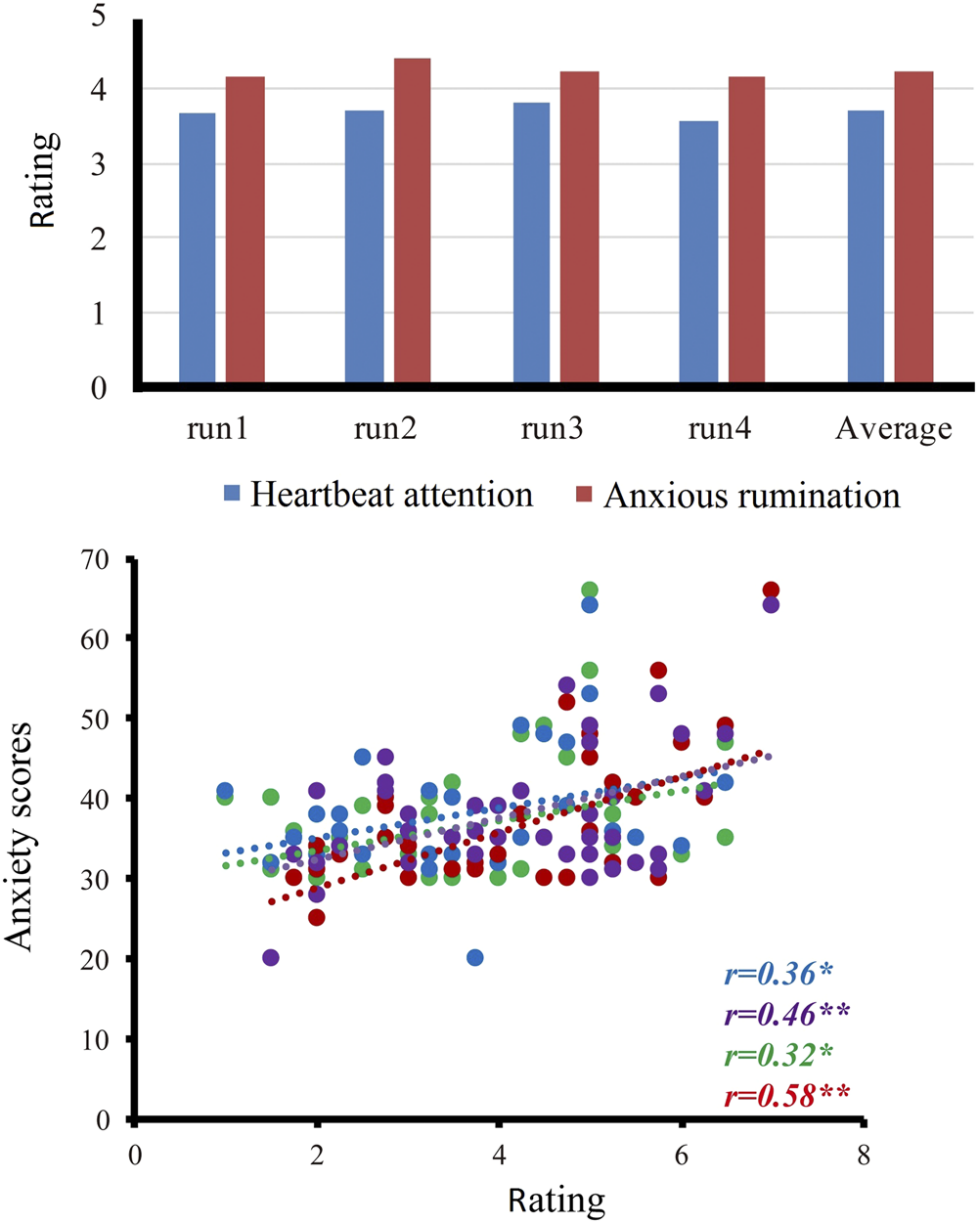
Subjects’ behavioral responses in the scanner. The upper panel shows the heartbeat awareness and anxious experience ratings in each run along with mean ratings. The lower panel shows that the ratings during heartbeat attention condition were significantly positively correlated with state (blue) and trait anxiety scores (green), and that the ratings during anxiety attention condition were significantly positively correlated with state (red) and trait anxiety scores (purple) (Pearson’s r). *p < 0.05; **p < 0.01.

The means, standard deviations (SD) and correlations of scores of Heartbeat Perception Task, anxiety and behavioral responses during the scanner task are presented in Table 1. The mean score in Heartbeat Perception Task for all subjects (N = 40) was M = 0.65, SD = 0.22 and ranged from 0.15 to 0.99. The distribution of the Heartbeat Perception Task score was congruent with the distributions reported in earlier studies on healthy samples^7,34^. Subjects’ behavioral responses in the scanner showed average ratings significantly exceeding the middle of the 7-point scale, indicating that they performed the tasks as instructed. The correlational analysis (Pearson’s r) revealed that both ratings during heartbeat attention condition and anxiety attention condition were significantly positively correlated with state and trait anxiety scores (Fig. 1). There was no significant association between Heartbeat Perception Task score and state anxiety (*r* = 0.17, *p* = 0.31), but a marginally significant correlation was found between the heartbeat perception score and the level of trait anxiety (*r* = 0.28, *p* = 0.08).

**Table 1:** Mean scores (SD) and correlations (Pearson) for performance on heartbeat perception, anxiety and intensity of interoception and anxiety in the scanner.

	Average (SD)	HP	TA	SA	H	A
HP	0.65 (0.22)		0.28	0.17	0.20	0.11
TA	38.25 (8.10)			0.94**	0.36*	0.46**
SA	36.68 (8.67)				0.32*	0.58**
H	3.69 (1.52)					0.04
A	4.23 (1.45)					

Abbreviations: SD: standard deviations; HP: heartbeat perception; TA: trait anxiety; SA: state anxiety; H: Heartbeat attention condition score in the scanner; A: Anxiety attention condition score in the scanner. *p < 0.05; **p < 0.01.

### Functional imaging data

**Figure 2:**
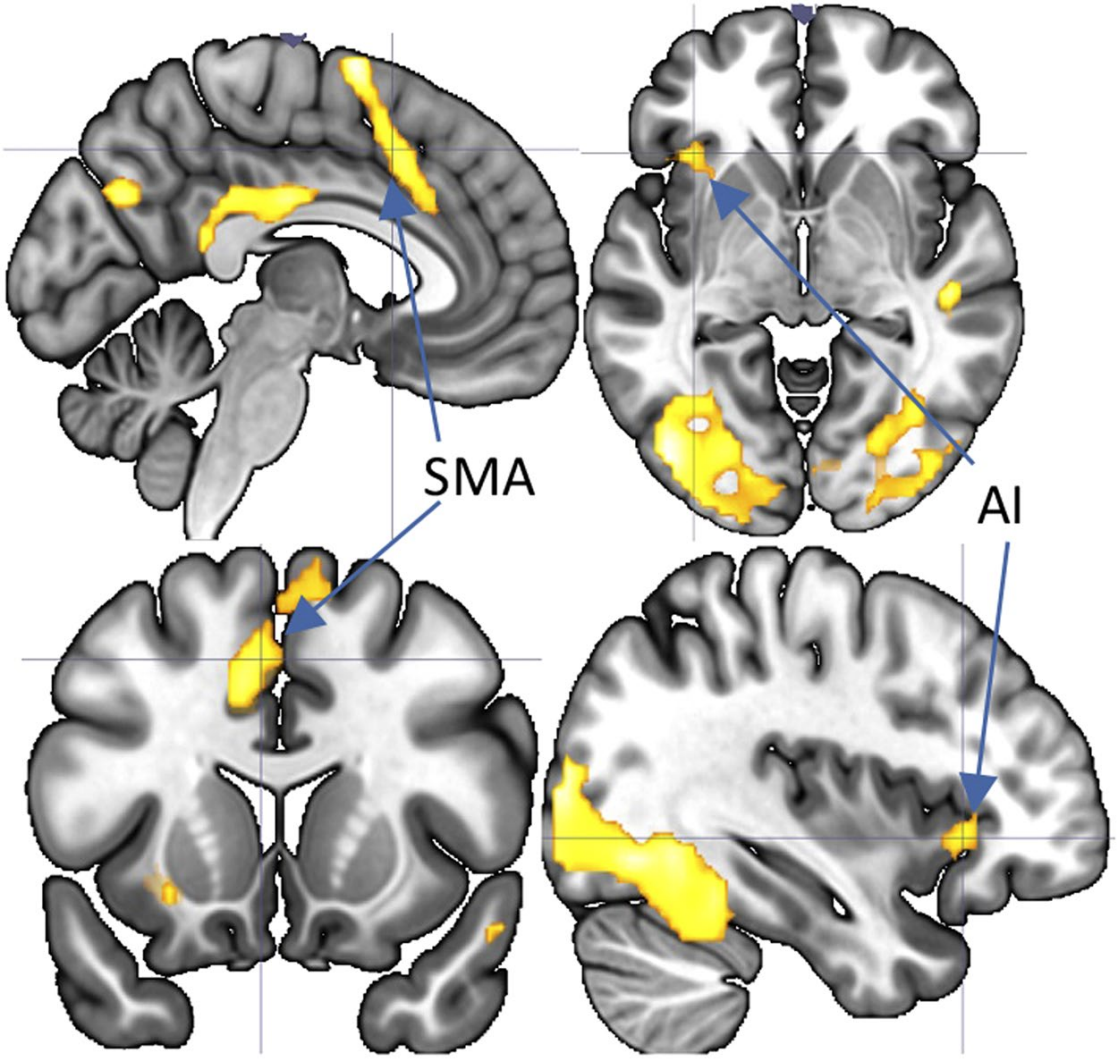
Commonly activated neural regions during the heartbeat attention condition and anxiety attention condition (*p*FWE < 0.01). SMA: supplementary motor area; AI: anterior insula.

To test the regions commonly associated with interoception and anxiety, we investigated the conjunctions between the cardiac interoception (heartbeat attention vs. exteroceptive attention) and anxiety (anxiety attention vs. exteroceptive attention) image contrasts. After controlling for age and sex, conjunction analysis revealed that the overlapping regions included the left anterior insular cortex, left supplementary motor area and left middle cingulate gyrus (Fig. 2).

**Figure 3:**
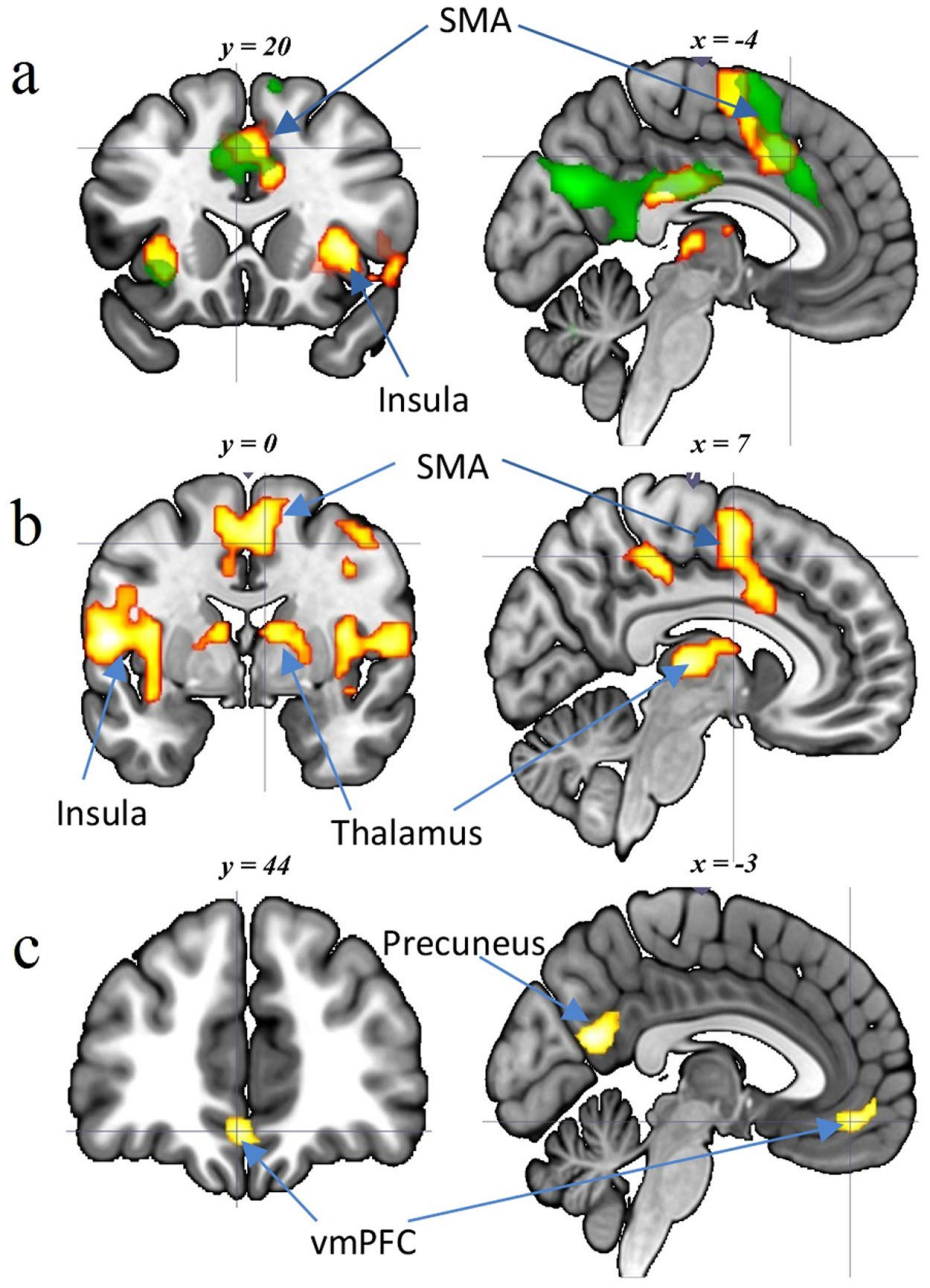
(**a**) Overlapping of cardiac interoception-related (orange, pFWE < 0.01) and anxiety-related regions (green, *p*FDR < 0.01). (**b**) Image contrast of heartbeat attention condition versus anxiety attention condition (*p*FWE < 0.01). (**c**) Image contrast of anxiety attention condition versus heartbeat attention condition (*p*FDR < 0.05). SMA: supplementary motor area; vmPFC: ventral medial prefrontal cortex.

Additionally, by investigating the interoception-related and anxiety-related brain regions using the Matlab toolbox xjView 8 (http://www.alivelearn.net/xjview8/index2.html), we found that heartbeat attention involved both anterior and posterior parts of the supplementary motor area and the insula while anxiety attention elicited activation of only the anterior part of the supplementary motor area and the insula (Fig. 3a).

Through comparing signal change between the heartbeat attention and the anxiety attention conditions, we found that cardiac interoception disproportionally activated the bilateral insular cortex, left precentral gyrus, bilateral supramarginal gyrus, bilateral thalamus and right supplementary motor area compared with anxiety attention condition (Fig. 3b).

At a more lenient threshold, anxiety attention condition disproportionally activated the ventral medial prefrontal cortex (vmPFC) and precuneus compared with heartbeat attention condition (p < 0.05, false discovery rate (FDR) corrected) (Fig. 3c). The above results are shown in Table 2.

**Table 2:** Anatomical locations and coordinates of activations.

Region	L/R	BA	Size (voxels)	*T* value	MNI coordinates		
					x	y	z
***Co-activity for interoception and anxiety***							
Supplementary motor area	L	6	322	6.02	−6	3	72
Middle occipital gyrus	L	19	990	5.68	−45	−81	−3
Cerebellum posterior lobe	R	37	711	5.41	39	−60	−27
	R	18	87	4.79	0	−69	−30
Cuneus	L	/	115	5.39	−9	−69	30
Cingulate gyrus	L	/	192	5.28	−3	−18	30
Superior temporal gyrus	R	/	33	5.20	48	−24	−3
Insula	L	48	73	4.36	−27	15	−9
***Activity related to interoception versus anxiety***							
Precentral gyrus	L	48	1548	9.83	−54	0	9
Thalamus	R	/	2198	9.60	12	−24	6
	L	/	53	9.04	−6	−21	6
Cingulate gyrus	L	24	391	9.21	−9	6	39
	R	31	105	8.08	12	−30	42
Middle frontal gyrus	L	45	129	8.68	−45	36	30
	R	6	35	6.70	42	3	54
Insula	L	48	116	8.26	−30	18	3
Precuneus	R	7	31	7.67	12	−69	42
***Activity related to anxiety versus interoception***							
Precuneus	L	31	14	5.41	−3	−63	21
Medial frontal gyrus	L	11	3	5.10	−3	45	−12
***Activity related to interoception versus exteroception***							
Thalamus	R	/	1283	9.15	9	−21	6
Insula	L	44	541	7.78	−42	3	9
Supramarginal gyrus	R	22	204	7.42	60	−36	21
	L	39	141	8.54	−51	−45	27
Cingulate gyrus	L	/	1247	9.49	−3	−30	27
	R	31	105	8.08	12	−30	42
Precuneus	R	7	120	7.36	12	−66	30
	L	7	35	6.84	−9	−69	33
Middle frontal gyrus	R	8	105	7.27	39	6	42
	L	6	75	7.28	−39	−3	57
Inferior parietal lobule	R	39	76	7.36	36	−51	45
	L	7	48	6.73	−33	−51	48

Note: L = left hemisphere, R = right hemisphere, BA = Brodmann area, MNI = coordinates referring to the standard brain of the Montreal Neurological Institute. For coactivity for interoception and anxiety, activity related to interoception versus anxiety and activity related to interoception versus exteroception, clusters of maximally activated voxels that survived statistical thresholding at *T* = 3.39, *T* = 6.11 and *T* = 6.11, respectively (*p*FWE < 0.01). For activity related to anxiety versus interoception, clusters of maximally activated voxels that survived statistical thresholding at *T* = 4.70 (*p*FDR < 0.05).

**Figure 4:**
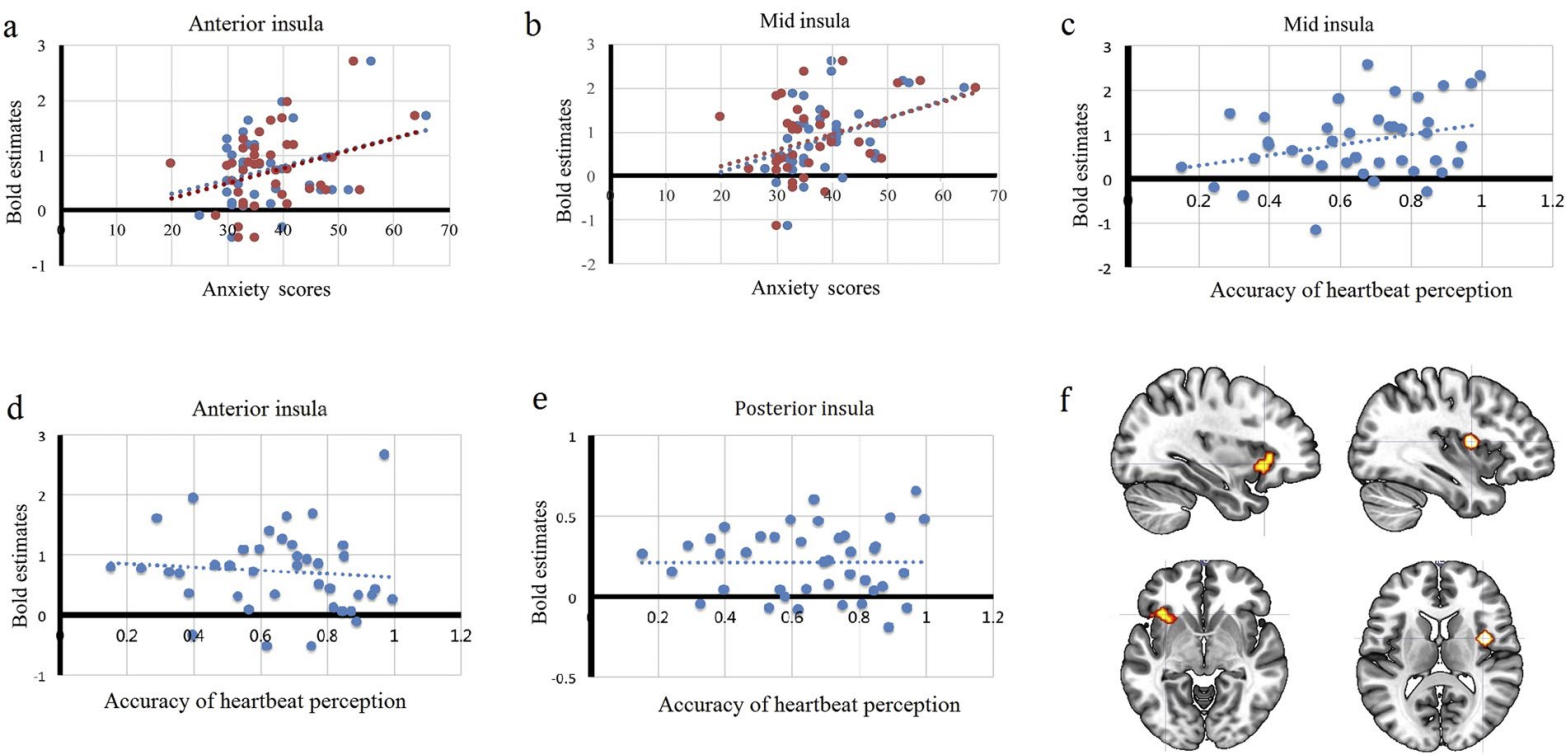
The correlations between insular activity during the heartbeat attention condition and behavioral data. (**a**) The activity of the left anterior insula was significantly positively correlated with state anxiety scores (blue dost; *r* = 0.33, *p* = 0.04) and trait anxiety scores (red dots; *r* = 0.33, *p* = 0.04). (**b**) The activity in the right mid-insula was significantly positively correlated with state anxiety scores (red dots; *r* = 0.39, *p* = 0.01) and trait anxiety scores (blue dots; *r* = 0.41, *p* = 0.01). (**c**) The activity in the right mid-insula was significantly positively correlated with accuracy of heartbeat perception (blue dots; *r* = 0.31, *p* = 0.05). (**d**) The activity in the left anterior insula was not correlated with accuracy of heartbeat perception (*r* = −0.09, *p* = 0.57). (**e**) The activity in the right posterior insula was not correlated with accuracy of heartbeat perception (*r* = 0.004, *p* = 0.98). (**f**) The locations of the anterior insula (left panel) and the mid-insula (right panel).

To investigate the precise activation pattern of the ROIs, we extracted the BOLD estimated value of the anterior insula and the mid-insula for heartbeat attention vs. exteroceptive attention contrast and anxiety attention vs. exteroceptive attention contrast (Fig. 4f). The left anterior insula was activated during both cardiac interoception and anxiety attention, while the right mid-insula was selectively activated during cardiac interoception. Additionally, the correlation analyses revealed that the BOLD estimate for the peak voxel within the left anterior insular cortex in heartbeat attention vs. exteroceptive attention contrast positively correlated with state (*r* = 0.33, *p* = 0.04) and trait anxiety scores (*r* = 0.33, *p* = 0.04) (Fig. 4a). The BOLD estimate for the right mid-insula in heartbeat attention vs. exteroceptive contrast was also positively related both to scores of state (*r* = 0.39, *p* = 0.01) and trait anxiety (*r* = 0.41, *p* = 0.01) (Fig. 4b), as well as heartbeat perception accuracy (*r* = 0.31, *p* = 0.05) (Fig. 4c). Moreover, the post-hoc exploratory analysis revealed no relationship between interoceptive accuracy and anterior insular activity (*r* = −0.09, *p* = 0.57) (Fig. 4d) or posterior insular activity (*r* = 0.004, *p* = 0.98) (Fig. 4e).

## Discussion

This study aimed to further clarify the neural correlates of cardiac interoception and the relationships between interoception-related insular activity, interoceptive accuracy and levels of anxiety in a healthy population sample. To investigate the neural activity underlying the focus on heartbeats and anxious events, a well-established fMRI paradigm was used. Interoceptive accuracy was assessed using Schandry’s^32^ Heartbeat Perception Task. Anxiety was measured using the State Trait Anxiety Inventory. The results of this study confirmed and expanded upon previous findings that related interoceptive attention and anxiety attention to selective activation along the insula in a caudal-to-rostral fashion, in accordance with the insula’s functional organisation^15^. Furthermore, the level of recorded activity in the left anterior insula during attentive focus on the heartbeat was positively associated to individual levels of state and trait anxiety. More importantly, the neural activity of the right mid-insula during heartbeat attention was not only positively related to individual heartbeat perception accuracy, but also to individual levels of state and trait anxiety. Taken together, these findings suggest that mid-insula may contribute to the interaction between cardiac interoception and anxiety.

The present study verified previous findings on the indiscriminant activation of the anterior insula during interoceptive processes and anxious experience^19,35,36^. These studies are in line with the James-Lange theory of emotion generation^18^, which argues that perception of physiological state is essential for emotional experience. A large body of evidence indicates that the anterior insula is of central importance to integrate internal bodily states, external stimuli and emotional experience^1,36,37^. Furthermore, neuroimaging studies have reported that uncertainty or ambiguity of subsequent emotion-laden events also caused anterior insular activation^38,39^. This further suggests that the anterior insula integrates visceral sensory information with information from the traditional sensory afferents^2^. This is especially the case when visceral information alone is ambiguous, or when an individual’s level of anxiety is high^40,41^. For example, a study examining the neural correlates of performance feedback in social anxiety found insular hyperactivation during presentation of feedback in highly socially anxious individuals. The authors suggested that insula plays a role in interoceptive processing and evaluation of the emotional salience of interoceptive stimuli^42^. Some researchers have proposed that higher level of anxiety was associated with heightened activity in the anterior insula during processing of certain kinds of salient stimuli^21^. Our current study, in accordance with this idea, found that the activity of the anterior insula was reflective of attending to bodily processes, and that anterior insular activity was also correlated with levels of state and trait anxiety. These findings indicate that anxious experience may be implicitly accompanied by perception of the physiological state. This idea is further explored in a review that examined the role of interoception in anxiety and anxiety disorders^43^. Future research on emotions would benefit from combination of neuroimaging, physiological indexes and psychological measurements.

Simmons and his colleagues, as well as several others have suggested that the dorsal mid-insula is crucial for interoception^15,23–26^. The present study adopted and revised the experimental paradigm from Simmons, *et al*.^15^ and found that, in contrast with the process of anxiety attention, the right dorsal mid-insula was uniquely activated when attending to one’s own heartbeat. Conversely, previous studies that considered the actual accuracy of heartbeat perception and its neural correlates only found activation of the anterior insula^7,19,22,28–31^. In light of these findings, it appears that merely attending to one’s heartbeat activates a more posterior area of the insula, whereas counting and detecting heartbeat, thereby increasing cognitive load, activates a more anterior area of the insula^3^. Recent studies involving isoproterenol and fMRI also have reported indications that the right mid-insula is a key node in the interoceptive network^44,45^. More importantly, several meta-analyses have suggested a particular role of the mid-insula for processing of interoception^10,14,27^. However, it should be noted that interoceptive processing more than likely involves a wide neural network, including among others the somatosensory cortex, insula, anterior cingulate cortex and vmPFC^4,22^. Accordingly, several recent studies have reported that the functional connectivity of interoceptive neural network varied with illness anxiety^46^ and tactile attention^47^. Therefore, future studies examining the neural bases underlying the relationship between interoception and anxiety at a network level are certainly needed.

In addition, our study found that the activity in the mid-insula during heartbeat attention was positively correlated with scores of Heartbeat Perception Task, and state and trait anxiety. Our findings expand upon previous studies and show that mid-insular activity is not only reflective of attending to the heartbeat^15,23–26^, but also correlates with objective measurements of cardioceptive accuracy. The results further support the proposal that interoception is essential to emotional experience^17,18^. Expanding on an earlier study that highlighted the role of anterior insula in the interaction between cardiac interoception and anxiety^22^, we suggest that mid-insula may also play an important role in the interoceptive representation of anxious experiences. For example, one study using interoceptive attention task with anorexia nervosa patients found that the activation of mid-insula during gastric interoceptive attention was related to levels of anxiety^26^. Moreover, recent studies found reduced binding of receptors for the inhibitory neurotransmitter γ- aminobutyric acid (GABA) in bilateral mid-posterior insula in PD patients^48^ and linked insular GABA concentration to interoceptive awareness^49^. In addition, proton magnetic resonance spectroscopy (^1^H-MRS) studies have found that state and trait anxiety were related to alterations of several neurotransmitter systems in healthy subjects^50–52^. A review has also emphasised the contribution of several neurotransmitters in the interactions within the limbic system involved in the regulation of stress^53^. Therefore, neurotransmission may be a promising target to examine the role of insular region and limbic system in the relationship between interoception and anxiety. More research is needed to further disentangle the respective roles and interactions of the mid-insula and the anterior insula in the processing of anxiety.

While our study primarily focused on insular activity, we additionally found that the bilateral ventral posterior thalami exhibited a strong association with heartbeat attention. The ventral posterior thalamic nucleus is considered the somatosensory relay nucleus that projects homeostatic afferent information to the primary somatosensory cortex^1^. Previous studies also reported activation of these areas during interoceptive attention^36^. In our present study, explorative analysis revealed that the right ventral posterior thalamus activity related to both the anterior insula and mid-insula activity in the heartbeat attention condition, which was not observed for the anxiety attention condition. These findings support the notion that the ventral posterior thalamic nuclei constitutes important node of the interoceptive neural network, and serves to monitor autonomous function^54^. Interestingly, a brain lesion study of a patient with bilateral insula damage reported that he was still able to experience emotion, likely due to the residual function of subcortical regions such as the brain stem and thalamus^55^. The exact role of the thalamic nuclei in the processing of cardiac interoception still needs further elucidation using fMRI and animal models.

In addition, the activation of the bilateral supramarginal gyrus was found to be significantly greater during heartbeat attention compared with anxiety attention. Although we did not make any specific predictions that the supramarginal gyrus would be involved in cardioceptive processing, this finding is in accord with previous research on the obsevation of the supramarginal gyral activity during interoceptive attention in both novices and expert interoceptive attention practitioners^56^. Their findings revealed that participants inexperienced in interoceptive attention showed activation of the supramarginal gyrus while the experts demonstrated a marked lack of activity in this region. They suggest two possible roles of the supramarginal gyrus in the interoceptive attention task; either it serves a crucial role in exteroceptive peripersonal information processing, or in attention reorientation/inhibition. However, past research on neural correlates of cardiac interoception did not report activation of the supramarginal gyrus during heartbeat attention^7,15,22^. Conversely, one study revealed that the supramarginal gyrus was involved more during emotional evaluation than during sensory evaluation^35^. Further research is needed to examine the exact role of the supramarginal gyrus in the processing of attention.

At a more lenient threshold, anxiety attention condition activated the vmPFC compared with heartbeat attention condition. This observation was in line with previous findings suggesting the important role of medial prefrontal cortex (mPFC) in modulating anxiety^57,58^. For example, using fMRI and ^1^H-MRS, a series of studies by Delli Pizzi *et al*. suggested that mPFC GABA content was central to the regulation of amygdala activity and variability of fronto-limbic effective connection, which were both related to anxiety processing^59–61^. In addition, previous studies also demonstrated neurotransmitter abnormalities within the vmPFC in anxiety disorders^62,63^. It is worth noting that the vmPFC is prone to susceptibility artifacts in standard gradient echo imaging. For example, the ventral frontomedian activations could not be detected during a Stroop colour-word task using gradient echo EPI sequence, however, when using a spin-echo EPI sequence, additional ventral frontomedian activations were observed^64^. In this regard, different acquisition sequences (e.g. spin echo sequences) are highly recommended for future studies to investigate the neural correlates of anxiety attention^65,66^.

There are a few minor limitations to our study. A first limitation is that we requested participants to focus on personal past anxious events. Therefore, a possible point of criticism could be that the emotional arousal during this anxiety attention task was not very high because past anxious events by their nature are generally no longer affecting or relevant to participants’ day-to-day life. This could provide an alternative explanation for the overall smaller neural activation during the anxiety attention task compared with the heartbeat attention task. However, the observed activity in the anterior insula and medial frontal gyrus during the anxiety attention task corresponds to previous findings on neural activity during anxiety^36^ and suggest that our manipulation was effective. A second potential limitation is that the scanner sound is an ongoing external stimulus present in all fMRI conditions. Consequently, it could be argued that the exteroceptive condition where participants were asked to attend to the sound is not the only condition during which participants could have done so. However, the same can be claimed about heartbeat, or memories of past anxious events: both were potentially accessible during other conditions as well. Participants’ behavioral responses in the scanner, with mean ratings higher than the middle of the scale, along with differences in brain activity between the three conditions indicate that participants did comply with instructions and genuinely focused their attention as required. A third potential limitation is that the sensor on the index finger could have made it possible for participants to have felt their own heartbeat in their index finger. While this cannot be entirely excluded, experimental instructions clearly emphasized that participants should only count their heartbeats by focusing on sensations in the chest area, and that they were prohibited from relying on peripheral sensations. Additionally, a growing number of studies have brought into question the reliability of the HPT, on the grounds that the participants may perform correctly based on beliefs about heartrate but without actually detecting any heartbeat sensations^67,68^. More reliable methods to assess individual differences in interoceptive accuracy are urgently needed.

In sum: previous studies have underscored the important role of the insular region in the interaction between anxiety and cardiac interoception, and this was further supported by our findings. We additionally found that activity in the mid-insula during heartbeat attention was associated with levels of state and trait anxiety in a sample of healthy individuals. These results suggest that perception of anxiety related physical sensations appears to be closely related to interoceptive signals arising from the cardiac system. Subsequent subjective interpretation of these cardiac sensations may in turn shape the emotional experience. This study provides a relevant contribution to the development of functional neuroanatomical models of interoception and anxiety, and may prove useful in developing more effective interventions for anxiety disorders.

## Methods

### Participants

Participants were 50 healthy university students at Southwest University, China who were the part of an ongoing project examining associations between brain imaging, creativity, and mental health. Data from some of these participants has been reported previously^69–71^. All participants were screened to confirm their healthy development by a self-report questionnaire before the scan. Exclusion criteria included a history of neurological or psychiatric disorders, head injury, exposure to psychotropic medications, impaired vision that is not corrected and pregnancy. The participants completed informed consent in accordance with the Declaration of Helsinki (2008). All participants were financially reimbursed for participating. Participants first completed the Heartbeat Perception Task^32^ and filled in questionnaires, and then proceeded to the fMRI machine where they performed a number of simple tasks (outlined in more detail later). All the participants had normal or corrected-to normal vision. Ten participants were removed from further analyses on account of extreme motion artifacts (>3 mm) during fMRI scanning. Thus, final data analysis included 40 participants (22 females; mean age = 22.6 years; SD = 0.88). Two of them were left-handed (When we excluded the left-handed participants, the results were not significantly affected). The study was approved by the Brain Imaging Center Institutional Review Board of Southwest University, in accordance with the Declaration of Helsinki. The methods were carried out in accordance with the approved guidelines.

### Heartbeat Perception Task

The Heartbeat Perception Task was based on the mental tracking method proposed by Schandry^32^. The Heartbeat Perception Task is a widely used paradigm for measuring one’s interoceptive accuracy. During the Heartbeat Perception Task, participants were seated in a comfortable chair in a sound-attenuated chamber while attached to the biofeedback equipment (NeXus-10 with BioTrace software) through a sensor on the index finger for recording blood volume, pulse and heart rate. After 3 minutes of baseline recording, participants were asked to count their own heartbeats silently without palpitating or without relying on any other peripheral sensations. Rest and perception periods were alternated as follows: rest (5–15 sec) - perception (25 sec) - rest (5–15 sec) - perception (35 sec) - rest (5–15 sec) - perception (45 sec) - rest (5–15 sec) - perception (60 sec), and the sequence of perception periods was counterbalanced between participants. The beginning and the end of the counting phases were signaled by a start and stop tone, respectively. After the stop signal, participants were required to verbally report the number of counted heartbeats. Participants were not informed about the length of any of the counting phases. The accuracy of heartbeat perception was quantified according to the following transformation: 1 − 1/4∑ [(| counted heartbeats − recorded heartbeats |)/recorded heartbeats]^72^.

### Questionnaires

The participants filled in the Chinese version of the State Trait Anxiety Inventory^33^ after performing the Heartbeat Perception Task to assess their levels of state and trait anxiety. The State Trait Anxiety Inventory is a reliable and well-validated self-report measurement of current (state) and dispositional (trait) anxiety. The scales for state and trait anxiety consist of 20 items each to which subjects are asked to answer to what degree the items describe their situational and dispositional perceptions on a 4-point Likert-type scale, where 1 indicates “Not At all” and 4 indicates “Very Much So”.

Additionally, the participants were also instructed to write about their anxious events during the past five years.

### Experimental design

We used a revised version of the task from Simmons, *et al*.^15^ to investigate the neural activity during heartbeat attention and anxiety attention. After verbal instructions about the fMRI task a brief training procedure followed, in order to make sure that the participants were familiar with the stimulus presentation and the rating method. Once participants were familiarized with the procedure, they performed the experimental task during four fMRI scanning runs with three intervals of 30 seconds, each run lasting 1 minute and 16 seconds. The task consisted of three different blocks in the following order: heartbeat attention condition, anxiety attention condition, and exteroceptive attention condition. The visual stimuli were back-projected onto a screen behind the participant’s head and viewed through a mirror mounted on the head-coil. In each of the fMRI scanning runs, three conditions were separated by a jittered inter-stimulus interval that varied randomly between 2–16 s (mean interval = 5.33 s) during which participants saw only a white fixation mark against a black background. Paradigm presentation and response recording were controlled using Eprime (www.pstnet.com).

During the heartbeat attention condition, participants viewed the Chinese word “heartbeat” presented respectively one time in each scanning run, in a white font against a black screen for 16 s. Participants were instructed that for the entire time during which the word remained onscreen, they should focus on the sensation of their own heartbeat. Immediately following each trial, participants were asked to rate how intensely they sensed their heartbeat during the preceding heartbeat attention period. Participants provided their ratings via a magnetic resonance-compatible handheld scroll-wheel that moved a cursor along a visual analog scale labeled from 1 to 7, with 1 indicating no sensation, and 7 indicating an extremely strong sensation. Participants had 4 seconds to complete their intensity ratings.

During the anxiety attention condition, similar to the heartbeat attention condition, participants saw the Chinese word meaning “anxiety” in each run for 16 s, when participants focused their attention on anxieties they had written down before scanning. Immediately following each trial, participants were required to rate how intensely they experienced anxiety during the preceding anxiety attention period on a scale from 1 to 7, with 1 indicating no anxiety, and 7 indicating an extremely intensive anxiety.

Similarly, during the exteroceptive attention condition, participants saw the Chinese word meaning “sound” presented in each run for 16 s, when participants attended to the sound of fMRI scanner. Immediately following each trial, participants were asked to rate how intense the sound was during the preceding exteroceptive attention period on a scale numbered from 1 to 7, with 1 indicating no sound, and 7 indicating extremely intensive sound.

### Data acquisition

All functional and structural magnetic resonance imaging (MRI) data were collected using a 3.0-T Siemens Trio MRI scanner (Siemens Medical, Erlangen, Germany) in the Southwest University Center for Brain Imaging. A T2* weighted single-shot gradient-recalled echo-planar imaging (EPI) sequence using Sensitivity Encoding (SENSE) technique in depicting blood oxygenation level depended (BOLD) contrast was applied to functional scanning. The EPI imaging parameters used were as follows: field of view (FOV) = 220 mm × 220 mm, acquisition matrix = 96 × 96, slices = 32, repetition time (TR)/echo time (TE) = 2000/30 ms, SENSE acceleration factor R = 2 in the phase encoding (anterior–posterior) direction, flip angle = 90°, thickness/slice gap = 3/1 mm, sampling bandwidth = 250 kHz, number of volumes = 225, scan time = 7 min 30 s. The EPI images were reconstructed into a 128 × 128 matrix, in which the resulting fMRI voxel volume was 3.4 × 3.4 × 3 mm^3^. The first 5 images before starting the experimental runs were discarded because of scanner equilibration effects. Moreover, a high-resolution T1-weighted image for anatomical reference of the fMRI analysis was acquired using a magnetization-prepared rapid gradient echo (MPRAGE) sequence with following parameters: slices = 176; slice thickness = 1.0 mm; TR/TE/TI = 1900 ms/2.52 ms/900 ms; flip angle = 9°; sampling bandwidth = 31.2 kHz; resolution matrix = 256 × 256; voxel size = 1 × 1 × 1 mm^3^; scan time = 4 min 58 s.

### Data preprocessing and analyses

Imaging preprocessing was performed using Statistical Parametric Mapping (SPM8) (http://www. fil.ion.ucl.ac.uk/spm) running on Matlab (The MathWorks, Inc). Following reconstruction, corrections for both slice time and head movement were applied to all echo-planar images, which were then normalized to the Montreal Neurological Institute (MNI) space determined by the T1-weighted anatomical images and spatially smoothed to an 8-mm full width at half-maximum Gaussian kernel. First-level analyses were performed to determine each participant’s voxel-wise activation during fMRI task through separately modeling the onsets of the heartbeat attention, anxiety attention and exteroceptive attention conditions as epochs convolved with a canonical hemodynamic response function (HRF). The 6 head motion parameters (3 translations, 3 rotations) were included as regressors of non-interest, and high-pass temporal filtering with a cut-off of 128 seconds was applied to remove low-frequency drifts in signal. The neural activity during different conditions was analysed for each participant, in the form of statistical parametric maps of distinct contrasts. Subsequent second-level group random effects analyses were performed to evaluate condition effects at the population-level.

To assess the neural activity difference between heartbeat attention and anxiety attention, the heartbeat attention condition was weighted against anxiety attention condition to form the contrast. The t-map was subsequently corrected for multiple comparisons using the familywise error rate (*p*FWE < 0.01).

Conjunction analyses were used to identify overlapping regions for heartbeat attention condition and anxiety attention condition^73^, which were defined by a conjunction of t-test with heartbeat attention >exteroceptive attention AND anxiety attention >exteroceptive attention, with FWE-corrected for multiple comparisons (*p*FWE < 0.01). Previous studies have suggested that age and sex may affect the interoceptive processing^74,75^. Therefor, we controlled for age and sex. Additionally, we applied cluster-size threshold of 30 voxels to the map in order to eliminate the possibility of small activity areas induced by spatial smoothing or resampling.

Primary representation of cortical regions of interest (ROI) was performed for further correlation analysis. Based on the previous research^12^ and the results from conjunction analysis in the current study, the anterior insula (6 mm sphere centered *x* = −27, *y* = 15, *z* = −9) was chosen as one of the ROIs. We also focused on the mid-insula (6 mm sphere centered *x* = 39, *y* = 0, *z* = 12), which was based on the identified region selectively for interoception by Simmons et al. (2012) and different brain regions between heartbeat attention and anxiety attention in this study. In the anatomical ROI analyses, mean time courses were extracted from each ROI using the region of interest extraction (REX) toolbox (http://web.mit.edu/swg/software.htm). For each participant, BOLD estimates were extracted separately for the heartbeat attention, anxiety attention, and exteroceptive attention conditions and a group-level *t*-test for those coefficients of each ROI was performed. Additionally, correlations between mean percent signal change of each ROI and behavioral data were also calculated.

## Data Availability

The datasets generated during and/or analysed during the current study are available from the corresponding author on reasonable request.
